# Association Between Prolonged Grief and Attitudes Toward Reconciliation in Bereaved Survivors of the Khmer Rouge Regime in Cambodia

**DOI:** 10.3389/fpsyt.2020.00644

**Published:** 2020-07-10

**Authors:** Nadine Stammel, Louisa Heinzl, Carina Heeke, Maria Böttche, Christine Knaevelsrud

**Affiliations:** ^1^Department of Clinical Psychological Intervention, Freie Universität Berlin, Berlin, Germany; ^2^Research Department, CENTER ÜEBERLEBEN, Berlin, Germany

**Keywords:** Cambodia, bereavement, grief, reconciliation, forgiveness, post-conflict, violent death, genocide

## Abstract

**Background:**

During the Khmer Rouge regime in Cambodia, about a quarter of the population died, resulting in many individuals losing close relatives. Still today, many individuals are suffering from the psychological consequences of these losses, which might also affect the process of reconciliation within the Cambodian society. The aim of this paper is therefore to investigate the association between symptoms of prolonged grief and attitudes toward reconciliation.

**Methods:**

A sample of 775 survivors of the Khmer Rouge regime who lost relatives during the conflict were interviewed about their losses and traumatic events, prolonged grief (PG; Complicated Grief Assessment Self-Report, CGA-SR), posttraumatic stress disorder (PTSD Checklist - Civilian Version) and attitudes toward reconciliation (Readiness to Reconcile Inventory, RRI).

**Results:**

A higher symptom severity of PG was significantly associated with readiness to reconcile even when controlling for other relevant variables (β = −0.22; p <.001). Persons who met caseness criteria for PG were significantly less ready to reconcile, t(773) = 5.47, p <.001, than persons who did not meet caseness for PG.

**Conclusion:**

PG seems to be a relevant mental health correlate of attitudes toward reconciliation. The results of the current study underline the importance of also considering PG with regard to the reconciliation process in Cambodia and possibly also in other post-conflict regions.

## Introduction

During the Khmer Rouge (KR) regime from 1975 to 1979, millions of Cambodians suffered grave human rights violations, including mass executions, forced labor, torture and starvation. The KR regime caused the death of about 1.7 million Cambodians—approximately one quarter of the Cambodian population at that time (1)—leaving behind a substantial number of bereaved and traumatized individuals.

Decades after the end of the KR regime, many Cambodians continue to suffer from mental health problems stemming from this time. About 30 years after the end of the KR regime, approximately 14% of KR survivors were suffering from posttraumatic stress disorder (PTSD; 2). A national representative survey conducted in 2011 found rates of 27.4% for anxiety and 16.7% for depression in the general population (3). Rates of psychiatric morbidity among survivors of the KR regime are even higher (4). Even though many survivors of war and violent conflict are confronted with the death of loved ones, the psychological consequences of these losses have only recently been addressed in war-torn societies. While the majority of survivors adjust to their loss after a certain period of grieving and continue with their lives, others show severe psychological symptoms connected to their grief over long periods of time (5). Over the last decades of research, this pathological form of grief has been given a variety of labels (e.g., “complicated grief (disorder)”, “disturbed grief”, and “pathological grief”), and was finally included as “prolonged grief disorder” (PGD) in the 11^th^ edition of the International Classification of Diseases (ICD-11; 6) in a new category for stress-related disorders. In the Diagnostic and Statistical Manual of Mental Disorders, fifth edition (DSM-5; 7), PGD was not formally included as a distinct clinical entity and was instead included as a condition for further research, termed “persistent complex bereavement disorder”. The ICD-11 and DSM-5 definitions of the disorder differ from each other in terms of symptoms and symptom duration requirement (6 vs. 12 months, respectively). This represents a significant challenge, as the use of diverse scales to assess PG has been shown to result in different prevalence rates (8). It may thus be the case that research findings obtained using one set of criteria may not apply to other definitions of PG (9). The current study is based on the criteria provided by Prigerson and colleagues (10). These authors originally introduced the term “prolonged grief disorder”, which also represents the most widely used term in the literature. The applied criteria were the most contemporary criteria at the time of our study. The defining feature of all sets of criteria for PGD is separation distress (i.e., persistent longing or yearning for the lost person) combined with cognitive, emotional and behavioral symptoms resulting in functional impairment. However, as the applied criteria differ slightly from the current PGD ICD-11 definition, we will use the term “prolonged grief (PG)” when describing the results of our study to avoid confusion with the current ICD-11 definition.

While around 10% of bereaved persons who have lost a significant other by natural death are estimated to develop PGD (11), the prevalence of PGD is usually higher in war-affected populations. Almost 35% of a sample of young adults who had lived through the war in Kosovo met criteria for PGD (12). In a sample in Rwanda, 8% met the criteria more than ten years after the loss of a loved person (5). One reason for the higher prevalence of PGD after violent conflicts might be the high numbers of violent and unnatural deaths that people have to face during war. A violent cause of death (i.e., death by homicide, suicide, or accident) has been recognized to have a higher association with PGD than natural deaths (5, 13, 14). A study investigating the construct of PGD in Cambodia also confirmed its validity (15).

The consideration of cultural aspects of the grieving process, such as its culturally accepted length, has been emphasized in the new ICD-11 criteria set (6). Moreover, in Cambodia, a consideration of religious and spiritual beliefs about death and grieving also seems to be of special relevance. Cambodian culture and social norms are influenced by Buddhism and folk religion, in which spiritual beings such as ghosts play an essential role (16). Many Cambodians believe that when somebody dies due to unnatural causes, this person cannot easily reincarnate, and their spirit instead remains in an “in-between” state (17, 18). Furthermore, if the person had thoughts of hate or was yearning for another person in the moment of death, this is thought to further make it difficult for that person to be reborn (16, 18). Another important aspect of mourning in Cambodia is Buddhist rituals, such as cremation and paying respect to the deceased with offerings at the temple, to ensure that the deceased can reincarnate. These rituals could not be carried out during the KR regime (16).

Due to the interplay of the historic circumstances of the genocide and cultural beliefs, it may be expected that Cambodians show a greater vulnerability to be affected by grief-related mental health problems (16). Findings indicate that the symptoms shown by Cambodian refugees are often shaped by their beliefs about the state of the person they have lost: Many individuals had frequent nightmares in which they saw their relatives who had died during the KR regime, and interpreted this as a sign that their loved ones had still not reincarnated (18, 19). Moreover, visual auras (as a symptom of migraine) were interpreted as evil spirits, which patients experienced as highly impairing and worrying (20). These cultural ideas and interpretations of specific symptoms may also shift a certain responsibility onto the bereaved persons, because they feel it is their duty to help with their loved one’s reincarnation by performing religious rituals for the deceased (16). Not being able to “make merit” for one’s ancestors can thus have negative consequences for one’s own next life (21) or one’s current life, if ancestors haunt survivors in the form of spirits (20). This can lead people to frequently perform rituals for the deceased and to be highly concerned about the lost person (17, 22), potentially rendering it more difficult to come to terms with the death and to focus on one’s own life again, even after several years.

Even after the regime of the KR ended, Cambodia remained strongly affected both by ongoing political instability and the long-term consequences of the KR (21, 23). In many villages in Cambodia, former victims and perpetrators continued to live side by side. Often, people did not know whether or what kind of crimes had been committed by former KR members in their communities, and feelings of anger and revenge still lingered under the surface (24). Although in Cambodian culture, anger is generally not shown in public (25), many survivors of the KR regime reported feelings of revenge and hatred toward the former KR (2, 26). For a long time, the atrocities committed by the KR were not publicly addressed in Cambodia. Only about 30 years after the end of the Khmer Rouge regime were the Extraordinary Chambers in the Courts of Cambodia (ECCC), also known as Khmer Rouge tribunal, established to investigate the atrocities committed by the KR. While the primary purpose of the ECCC is to seek retributive justice, it was also expected to contribute to national reconciliation (27).

The awareness and importance of reconciliation in war-torn societies has grown in the past decades. Reconciliation between former opponents has become a key concept for sustainable peace activities in post-conflict societies. Although there is not yet a consensus definition of post-conflict reconciliation, most authors agree that reconciliation is a long-term process aiming at (re)building relationships between formerly hostile groups (e.g., 28, 29). Reconciliation also involves letting go of hatred and desire for revenge (29) as well as changing stereotypic beliefs about both the adversary group and one’s own group (30, 31). This includes being able to differentiate between members of the adversary group, i.e., perceiving them as individuals and human beings, rather than generalizing them as “evil”. It is assumed that coping with conflict-related traumatic events can have positive benefits for victims’ openness to reconciliation with former perpetrators, for example through legal-political measures such as war tribunals or truth commissions (32). This is partly supported by empirical research showing that persons with a poorer mental health outcome are generally less open to reconciliation and forgiveness (33–35). Studies have mostly focused on symptoms of PTSD (PTSD; 33, 34) when studying attitudes toward reconciliation or forgiveness and mental health, but to a lesser extent have also investigated other mental health outcomes such as depression and anxiety (5, 36). To our knowledge, the association of PGD with attitudes toward reconciliation or forgiveness in post-conflict societies has not yet been empirically investigated. However, it seems plausible that persons who are still suffering from conflict-related loss(es) will be less open to reconcile with those whom they hold responsible for the death of their loved ones.

Generally, evidence on risk and facilitating factors of reconciliation in post-conflict settings is very limited, as study findings are inconsistent and longitudinal data are still missing. Attitudes toward reconciliation and forgiveness do not seem to be directly related to the victims’ traumatic experiences, such as the number of traumatic events experienced (33, 37) but rather seem to be related to mental health consequences of traumatic events. However, research findings are not consistent. Furthermore, research on sociodemographic characteristics such as gender and educational level has yielded inconsistent results. While in some studies, a higher level of education was related to more openness to reconciliation (38, 39), other studies found the opposite to be the case (34, 40). Findings also differ with respect to whether one gender is associated with being more or less open to reconciliation (36, 41, 42).

In summary, empirical evidence on reconciliation and mental health, characteristics of PG as well as cultural aspects about loss and the grieving process in Cambodian society suggest an interplay between PG and the process of reconciliation in Cambodia. The main aim of this paper was therefore to investigate associations between attitudes toward reconciliation and PG symptoms in bereaved survivors of the KR regime. In particular, we wanted to find out (1) whether bereaved victims of the KR with PG, who lost at least one family member during the KR regime, differ in terms of their attitudes toward reconciliation from those without PG; (2) whether readiness to reconcile is related to PG symptoms and loss- and trauma-specific variables as well as sociodemographic characteristics; and (3) whether PG symptom severity is associated with readiness to reconcile beyond PTSD symptom severity as well as loss- and trauma-specific and sociodemographic variables.

Based on research on attitudes toward reconciliation in post-conflict settings and other trauma-related mental health problems described above, we expected that persons with PG would be less ready to reconcile than those without PG and that PG symptom severity would be negatively associated with readiness to reconcile, also when controlling for other relevant variables (number of lost close family members, trauma exposure, PTSD symptom severity, and sociodemographic characteristics).

## Methods

### Sample and Procedure

Participants were recruited between October 2008 and May 2009 through a convenience sampling approach in 19 out of 24 provinces in Cambodia. Potential participants were either contacted with the help of local non-governmental organizations or directly in their villages. Inclusion criteria for participants were date of birth before 1975 and considering oneself as a victim of the KR regime. For this analysis, we only included participants who had lost at least one (close or distant) family member during the KR regime. Of 1593 persons who were contacted, 796 did not wish to participate and a further 22 persons had to be excluded due to a lack of important data (missing or ambiguous information regarding the loss to which participants’ answers referred). The final sample thus comprised 775 individuals. The same data set was used in a previous publication on PG in Cambodia (43); the present analysis is thus secondary in nature.

After participants had been fully informed about the study and the voluntary nature of participation, written informed consent was obtained. Given the high illiteracy rate, the self-rating questionnaires were administered in the form of face-to-face interviews. The interviews were conducted in the local Khmer language by eight local Cambodian interviewers who held a B.A. degree in psychology and had previously participated in a two-week training course on the assessment of relevant concepts and use of the questionnaire measures. To maintain the quality of the interviews, the interviewers were supervised on a regular basis. Participation in the study was voluntary; no financial compensation was offered, but participants received a small gift consisting of a traditional Cambodian scarf “Krama” and a piece of soap (worth the equivalent of USD 1.5) after the end of the interview. Psychological counseling by telephone was offered to all participants in case of need. The study protocol was approved by the University of Constance Review Board and the Cambodian Ministry of the Interior.

### Measures

Symptoms of PGD were assessed using the Complicated Grief Assessment Self-Report (10). If participants reported the loss of more than one family member during the KR regime, they were asked to specify whose death was most difficult to cope with and to relate their answers to this person. The CGA-SR is a self-report questionnaire that assesses the frequency of separation distress and whether this separation distress disrupts one’s everyday life (Criterion A). Criterion B contains eight cognitive, behavioral and emotional symptoms (difficulties accepting the death, trusting people, moving on, feeling bitter over the death, feeling numb or difficulty connecting with others, feeling that life is empty or meaningless, as well as future holds no meaning or purpose and feeling on edge, jumpy or easily startled), measured on 5-point Likert scales from 1 to 5. Criterion C contains the question whether the symptoms lead to impairment in functional areas, and criterion D assesses whether the symptoms have already lasted for at least six months. Caseness for PG was indicated when a person fulfilled at least one of the two criterion A symptoms, as well as the C and D criteria, and stated being affected by at least four of the eight symptoms in criterion B to an extent of at least four points. Symptom severity of PG was measured by summarizing the values of symptom items with Likert scales. Higher scores indicate a higher symptom severity, with a minimum score of 9 and a maximum score of 45. Internal consistency of the CGA-SR in the current study was α = .82.

Readiness to reconcile was measured using the Readiness to Reconcile Inventory (RRI) (44). The RRI is a 13-item self-rating instrument assessing individual openness to reconcile with members of the former adversary group, thereby focusing exclusively on the perspective of the victims. In this study, the term “former adversary group” was substituted with “former Khmer Rouge”. The RRI consists (41) of three subscales: 1. openness to positive relationships with former members of the KR (six items; example item: “I would like us and the former Khmer Rouge to live in peace together”), 2. absence of feelings of revenge toward former members of the KR (five items; example item: “I have feelings of revenge when I think of the former Khmer Rouge”), and 3. differentiation ability (two items; example item: “The majority of the former Khmer Rouge are good people”). The latter describes the ability to differentiate between individual perpetrators and the group of former members of the KR. All items of subscale 2 (absence of revenge) are negatively coded. Participants are asked to indicate their level of agreement with each item on a 5-point Likert scale from 1 (*strongly disagree*) to 5 (*strongly agree*), with higher scores indicating greater readiness to reconcile. The factor structure and the internal consistency of the RRI and its pilot version were confirmed in several studies with different samples (37, 44, 45). Cronbach’s alpha for the RRI total scale in the current study was α =. 87. For the subscales, Cronbach’s alphas were as follows: openness to interactions, α = .85; absence of revenge, α = .87; differentiation ability, α = .72.

Symptoms of PTSD were measured using the PTSD Checklist‒Civilian Version (PCL-C; 46), a widely used 17-item standardized self-report rating scale that corresponds to the DSM-IV diagnostic criteria for PTSD. Participants were asked to rate each item on a 5-point Likert scale from 1 (*not at all*) to 5 (*extremely*) referring to the past month. The sum score was used as an indicator of PTSD symptom severity. The Cambodian version of the PCL-C has shown excellent reliability in earlier studies (2, 38). In the present study, the internal consistency was *α* = 0.88.

Potentially traumatic events were assessed using an adjusted checklist based on the first part of the Harvard Trauma Questionnaire (47) and the Posttraumatic Diagnostic Scale (48). Additionally, seven traumatic events specific to the Khmer Rouge regime (e.g., forced labor and forced marriage) were included. In total, 29 types of traumatic experience were assessed, creating an index of overall trauma exposure. Event types were rated as potentially traumatic if participants indicated having experienced or witnessed them. If the event included the death of another person, also having heard about such an event was rated as potentially traumatic.

In addition to traumatic events, loss-related questions were administered, including how many losses within one’s close family (i.e., spouse, parent, child, and sibling) as well as within one’s distant family (i.e., grandparent, grandchild, uncle/aunt, nephew/niece, cousin, and in-law) were experienced during the KR regime.

All questionnaires were translated from English into Khmer by bilingual psychologists, and subsequently back-translated by interpreters who were unfamiliar with the original English version in order to verify their correspondence. Discrepancies between the original and back-translated versions were discussed, and wherever necessary, the Khmer version was adjusted. Recommendations for cross-cultural adaptation were considered throughout the translation process (e.g., 49).

### Statistical Analysis

A first screening of the data showed that only a small amount of data was missing (0.29%). Missing values within the CGA-SR, the PCL-C, and the RRI were replaced using the expectation-maximization method (EM; 50). T-tests were calculated to determine whether people who met caseness criteria for PG according to the CGA-SR and those who did not differed in their attitudes toward reconciliation. This was done for the RRI total scale as well as for the RRI subscales (openness to interactions, absence of revenge, differentiation ability). Correlation analysis was performed to determine associations of the RRI and its subscales with the investigated variables. Hierarchical stepwise multiple linear regression was applied to investigate whether PG symptom severity (using the sum score) can explain variance in the readiness to reconcile beyond sociodemographic, loss- and trauma-specific variables, as well as PTSD symptom severity. The demographic variables gender, age, and education were entered in the first step of the regression analysis. The number of traumatic event types and the number of losses within the close family were entered in the second step, PTSD symptom severity was entered in the third step, and PG symptom severity was entered in the final step. To better understand how PG symptoms and reconciliation are associated, the regression analysis was also repeated for the subscales of the RRI as outcome variables. Relevant assumptions for multiple regression analysis were tested (linearity, multicollinearity, independency of residuals, and homoscedasticity) and did not reveal any violation. The analysis was conducted using SPSS 25.

## Results

### Sociodemographic Characteristics

Almost two thirds of the sample were female, and the mean age was 56 years. On average, participants had spent four years in formal education. Most of the participants identified themselves as Khmer and Buddhists. About two thirds of the sample were married and about 30% were widowed. The detailed sociodemographic characteristics of the sample are shown in **Table 1**.

**Table 1:** Sociodemographic characteristics.

Characteristic	*N* = 775
Sex = female, *n* (%)	498 (64.3)
Age, *M* (*SD*)	56.7 (10.3)
Years of formal education, *M* (*SD*)	4.1 (3.6)
Self-identity	
Khmer, *n* (%)	678 (87.5)
Cham, *n* (%)	45 (5.8)
Chinese, *n* (%)	15 (1.9)
Other, *n* (%)	37 (4.8)
Religion	
Buddhist, *n* (%)	702 (90.6)
Muslim, *n* (%)	58 (7.5)
Other, *n* (%)	15 (1.9)
Marital status	
Married, *n* (%)	497 (64.1)
Widowed, *n* (%)	231 (29.8)
Divorced, *n* (%)	25 (3.2)
Single, *n* (%)	11 (1.4)
Other, *n* (%)	11 (1.4)

### Loss- and Trauma-Specific Characteristics, PG, and Readiness for Reconciliation

Participants stated that they had lost on average 6.4 relatives during the KR regime, of whom on average 3.4 were part of the close family and 3.2 were part of the distant family. When participants were asked which loss was the most difficult to cope with, and upon which their answers on the CGA-SR were thus based, 14.6% (n = 113) reported the loss of a spouse, 5.3% (n = 41) the loss of a child, 34.1% (n = 264) the loss of a parent; 28.6% (n = 222) the loss of a sibling, and 17.4% (n = 135) the loss of a distant relative.

Participants reported on average 14 different traumatic event types. The five most frequently reported traumatic event types were lack of food or water (95.6%; n = 741), forced labor (94.1%; n = 729), forced separation from family members (81.1%; n = 629), deportation/forced displacement (79.9%; n = 619), and life-threatening illnesses (70.5%; n = 547).

Of the whole sample, 111 participants (14.3%) met caseness criteria for PG as measured by the CGA-SR. The mean sum score of the CGA-SR across the whole sample was 20.99. The mean sum total score of the RRI was 32.28. The mean sum scores of the RRI subscales were 13.00 for openness to interactions, 15.68 for absence of revenge and 3.60 for differentiation ability, respectively.

The detailed descriptive data on losses, traumatic event types, PTSD, PG, and readiness for reconciliation are shown in **Table 2**.

**Table 2:** Descriptive data on losses, traumatic events, PG symptom severity, PTSD symptom severity and readiness to reconcile.

	***M***	***SD***	**Answer Range**	
			**Min**	**Max**
Number of losses (in total)	6.40	6.79	1	59
Number of losses (close family)	3.35	3.37	0	27
Number of losses (distant family)	3.16	6.18	0	50
Number of traumatic event types	14.01	3.84	3	25
PG symptom severity^1^	20.99	7.37	9	45
PTSD symptom severity^2^	30.10	11.35	3	75
RRI total score	32.28	10.94	13	62
RRI Openness to Interactions	13.00	5.94	6	30
RRI Absence of Revenge	15.68	5.99	5	25
RRI Differentiation Ability	3.60	1.64	2	9

PG, Prolonged Grief; PTSD, Posttraumatic Stress Disorder; RRI, Readiness to Reconcile Inventory.

^1^Complicated Grief Assessment Self Report (CGA-SR); ^2^ PTSD-Checklist Civilian Version (PCL-C).

### Differences Between Participants With and Without PG Regarding Readiness for Reconciliation

To ascertain whether people who meet caseness criteria for PG are less ready to reconcile than persons who do not meet caseness criteria, t-tests for the RRI total score as well as for the RRI subscales were performed. For this purpose, the sample was divided into persons who fulfilled the criteria for PG according to the CGA-SR and those who did not.

As shown in **Table 3**, participants meeting caseness criteria for PG were significantly less ready to reconcile than participants who did not meet caseness for PG according to the CGA-SR. This was the case for the RRI total score as well as for all RRI subscales. These differences had a medium effect size for the RRI total scale as well as for the subscale absence of revenge. For openness to interactions as well as for differentiation ability, the effect sizes of the differences were small.

**Table 3:** T-tests with p-values, confidence intervals, and Cohen**’**s d.

Outcome	PG^1^ (*n* = 111)		No PG^1^ (*n* =664)		Average difference (95% CI)	*t* (*df*)	*d*
	*M*	*SD*	*M*	*SD*			
Readiness to Reconcile^2^	27.12	10.34	33.15	10.81	6.03 (3.86–8.19)	*t* (773) = 5.47***	0.57
Openness to Interactions^2^	10.98	5.11	13.33	6.01	2.35 (1.29–3.41)	*t* (165.14) = 4.37***	0.42
Absence of Revenge^2^	12.91	6.31	16.15	5.81	3.23 (2.05–4.42)	*t* (773) = 5.35***	0.53
Differentiation Ability^2^	3.23	1.60	3.67	1.64	0.44 (0.11–0.77)	*t* (773) = 2.63**	0.27

PG, prolonged grief; CI, confidence interval; d, Cohen’s d; ^1^ as measured with CGA-SR; ^2^ as measured with the RRI, **p < .01, ***p < .001 (two-tailed p-values).

### Associations of Readiness to Reconcile With PG and Sociodemographic and Loss- and Trauma-Specific Variables

To analyze associations of PG with other variables, the sum score of the CGA-SR was applied. Correlations of PG symptom severity, readiness to reconcile and the other variables included in this analysis can be found in **Table 4**. To investigate whether PG symptom severity can explain variance in the readiness to reconcile beyond PTSD symptom severity, sociodemographic, and loss- and trauma-specific variables, a stepwise hierarchical regression analysis was performed with the RRI total score as dependent variable. The same analysis was performed with the three RRI subscales, respectively. Sociodemographic characteristics (gender, age, education) were entered into the analysis first, followed by loss- and trauma-specific variables (number of losses within the close family, number of traumatic event types) in the second step, PTSD symptom severity in the third step, and PG symptom severity in the fourth step. Detailed data of the stepwise regression analysis of the RRI total score are depicted in **Table 5**.

**Table 4:** Pearson correlation coefficients.

	Readiness to Reconcile^1^	Openness to Interactions^2^	Absence of Revenge^2^	Differentiation Ability^2^	Male gender	Age	Education (years)	Number of losses (close family)	Number of traumatic event types	PTSD symptom severity^3^	PGD symptom severity^4^
Readiness to Reconcile^1^	1										
Openness to Interactions^2^	0.85***	1									
Absence of Revenge^2^	0.82***	0.42***	1								
Differentiation Ability^2^	0.59***	0.52***	0.29***	1							
Male gender	0.26***	0.34***	0.09*	0.16***	1						
Age	0.01	−0.12**	0.13***	0.02	−0.01	1					
Education (in years)	0.17***	0.25***	0.03	0.11**	0.40***	−0.22***	1				
Number of losses (close family)	−0.12**	−0.06	−0.13***	−0.9*	−0.08*	0.18***	−0.05	1			
Number of different traumatic events	−0.03	0.11**	−0.17***	0.03	0.09*	−0.07	0.09*	0.13***	1		
PTSD symptom severity^3^	−0.31***	−0.22***	−0.33***	−0.10**	−0.25***	−0.05	−0.12**	0.12**	0.18***	1	
PG symptom severity^4^	−0.34***	−0.23***	-0.35***	−0.17***	−0.23***	−0.02	−0.10**	0.18***	0.11**	0.61***	1

PTSD, Posttraumatic stress disorder; PG, Prolonged grief disorder; ^1^RRI; ^2^Subfactor of the RRI; ^3^PCL-C; ^4^ CGA-SR; *p <.05, **p < .01, ***p < .001 (two-tailed p-values).

**Table 5:** Hierarchical multiple regression analysis predicting readiness to reconcile (N = 774).

	Variables	B (SE B)	*β*	R^2^	ΔR^2^
Step 1	Male gender	5.21 (0.87)	0.23***	0.074	0.074***
	Age	0.03 (0.04)	0.03		
	Education (years)	0.26 (0.12)	0.08*		
Step 2	Male gender	5.09 (0.87)	0.22***	0.087	0.013**
	Age	0.05 (0.04)	0.05		
	Education (years)	0.27 (0.12)	0.09*		
	No. of losses (close family)	−0.32 (0.12)	−0.10**		
	No. of traumatic event types	−0.13 (0.10)	−0.05		
Step 3	Male gender	3.65 (0.86)	0.16***	0.144	0.057***
	Age	0.03 (0.04)	0.03		
	Education (years)	0.24 (0.11)	0.08*		
	No. of losses (close family)	−0.25 (0.11)	−0.08*		
	No. of traumatic event types	0.01 (0.10)	0.00		
	PTSD symptom severity^1^	−0.24 (0.03)	−0.25***		
Step 4	Male gender	3.33 (0.85)	0.15***	0.173	0.029***
	Age	0.03 (0.04)	0.03		
	Education (years)	0.23 (0.11)	0.08*		
	No. of losses (close family)	−0.17 (0.11)	−0.05		
	No. of traumatic event types	0.00 (0.10)	0.00		
	PTSD symptom severity^1^	−0.12 (0.04)	−0.13**		
	PG symptom severity^2^	−0.32 (0.06)	−0.22***		

PTSD, Posttraumatic stress disorder; PG, prolonged grief; ^1^PCL-C; ^2^CGA-SR; *p < .05, **p < .01, ***p < .001 (two-tailed p-values).

#### RRI Total Score

Sociodemographic characteristics explained 7.4% of the variance in readiness to reconcile. Of the investigated sociodemographic variables, gender and education were significantly associated with readiness to reconcile, with males as well as more educated persons showing higher scores for readiness to reconcile. The inclusion of loss- and trauma-related variables in step 2 explained 1.3% of additional variance. While there was no significant association between trauma exposure and the RRI total score, the number of losses within the close family was associated with readiness to reconcile in the second step (more losses were associated with less readiness to reconcile), but this association lost its significance when the PG symptom score was entered in the fourth step. PTSD symptom severity was significantly negatively associated with readiness to reconcile. The inclusion of PG symptom severity in the final step explained significantly more variance than did the model without PG symptom severity (2.9% of incremental variance), with a regression coefficient of β = −.22, p <.001. Thus, PG symptom severity was negatively associated with readiness to reconcile even after controlling for the other mentioned variables. The final model explained 17.7% of the variance in the RRI total score.

#### RRI Subscales

PG symptom severity was significantly negatively associated with all RRI subscales (β = −.12, p <.01; β = −.24, p <.001; β = −.14, p <.01). PTSD symptom severity, which was entered in step 3, accounted for significant additional variance in RRI-openness to interactions (ΔR^2^ = 0.02, p <.001) as well as in RRI-absence of revenge (ΔR^2^ = 0.07, p <.001) but not in RRI-differentiation ability (ΔR^2^ = < 0.01, p = .12). The inclusion of PG in the final step explained significantly more variance in all subscales than the models without PG symptom severity (0.9%, 3.4%, and 1.3% of additional variance), respectively. The final regression models explained between 5.4% and 17.9% of the variance in the subscales. RRI-openness to interactions was significantly associated with male gender, younger age and higher educational level as well as with a higher trauma exposure and lower PTSD and PG scores. RRI-absence of revenge was significantly related to older age, fewer losses within the close family, and less trauma exposure, as well as with lower PTSD and PG scores. RRI-differentiation ability was significantly associated with lower PG score and male gender, but not with the PTSD symptom score. The three stepwise regression analyses of the RRI subscales can be found in the supplementary material (**Supplementary Table 1**).

## Discussion

The present study examined associations of PG with attitudes toward reconciliation 30 years post loss in a sample of bereaved survivors of the KR regime who had lost at least one family member during the KR regime. Most participants had lost more than one relative: On average, the loss of approximately six family members was reported, including about three close family members. Even three decades after experiencing the loss(es), around 14% of the participants met criteria for PG according to the CGA-SR.

The results confirm our hypothesis that bereaved victims who met caseness criteria for PG were significantly less ready to reconcile compared to those who did not fulfill these criteria. This pattern was also found for all three subscales of the RRI. Thus, persons with probable PG were significantly less open to interacting with former members of the KR, had more feelings of revenge toward former members of the KR, and were less able to differentiate between individual perpetrators and members of the KR as a whole. As hypothesized, PG symptom severity was negatively associated with readiness to reconcile, also when controlling for PTSD, loss- and trauma-specific variables, as well as sociodemographic variables that were also expected to be associated with attitudes toward reconciliation. PG symptom severity explained around 3% of unique variance in readiness to reconcile and between 0.9% and 3.4% of additional variance in the RRI subscales. PG symptom severity was more strongly related to readiness to reconcile than any other analyzed variable.

Even though PG symptom severity explained a rather small amount of additional variance in readiness to reconcile, it should be considered that PTSD symptom severity was controlled for in a prior step, and that PTSD has so far been investigated as a main mental health correlate of attitudes toward reconciliation. As PTSD and PG correlate highly in the context of violent loss (51), it is not surprising that PG symptoms did not explain a large amount of additional variance. Moreover, in the current study, the association between PG and PTSD symptoms was rather strong (r = .59). The fact that in our study, PG symptom severity was the strongest predictor of readiness to reconcile, beyond PTSD as well as trauma- and loss-related variables, speaks for the importance of PG in this regard. Our results thus suggest that PG should be considered as a mental health correlate of reconciliation besides PTSD and other mental health outcomes.

Interestingly, the number of losses within the close family was significantly related to readiness to reconcile, but this association lost its significance when PG symptom severity was entered into the regression analysis. Only in one subscale of the RRI, namely, absence of revenge, was the number of losses within the close family still significantly related in the final regression model, albeit to a smaller extent than PG symptom severity. This implies that it is not the losses *per se*, but rather the psychological reaction to the losses which seems to be related to attitudes toward reconciliation with the former KR. Nevertheless, it is possible that qualitative aspects of losses are particularly linked to attitudes toward reconciliation rather than quantitative aspects such as the number of losses within the close family. Trauma exposure was not related to the total score of the RRI inventory, but was associated with the RRI subscales openness to interactions and absence of revenge. As expected, a higher trauma exposure was associated with more feelings of revenge. Surprisingly, this association was reversed for openness to interactions, with a higher trauma exposure being related to more openness to interactions. Most previous studies conducted in post-conflict settings did not find a relationship between trauma exposure and attitudes toward reconciliation (33, 35, 52), which is in line with the results of the current study. However, our results also suggest a more complex interaction between trauma exposure and different aspects of reconciliation, highlighting the need for more research in this regard. As in previous studies, higher levels of PTSD symptoms were associated with less readiness to reconcile. This pattern was also found for the RRI subscales, with the exception of RRI-differentiation ability, where no significant association was found. Among the investigated sociodemographic variables, we found that males and participants with a higher level of education were more open to reconciliation, although there were some variations in the subscales.

The study results imply that PG could play a similar role to PTSD as a mental health-related correlate of reconciliation attitudes and that PG should be considered with regard to the reconciliation process in Cambodia and possibly also in other post-conflict settings. These results are in line with previous research showing that persons with poorer mental health outcomes were generally less ready to reconcile (33, 34, 35). However, previous studies primarily focused on PTSD when investigating attitudes toward reconciliation and mental health. To our knowledge, no other empirical study has yet focused on the relationship between PG and readiness to reconcile or forgiveness in post-conflict settings. Perhaps unsurprisingly, for persons still struggling with conflict-related loss of a loved one, being open to reconciliation with the former perpetrators is very difficult. It might be that persons who are still suffering from the conflict-related loss do not feel entitled to be open to reconciliation or feel that they would betray the deceased if they did reconcile. Especially when thoughts about the deceased and grieving occur on an everyday basis, as is common in PG, moving beyond negative feelings toward those who are held responsible for the death of their loved ones is highly challenging. Persistent feelings of anger about the loss and a reluctance to socialize are possible aspects of PGD (53). Therefore, symptoms of PG might reduce willingness to interact with former perpetrators and increase negative feelings toward them. Instead, the symptoms might strengthen resentment against perpetrators.

A further binding element of PG and reconciliation after conflict might be the inability to find sense and meaning in what has happened. In particular, people bereaved by homicide have difficulties finding any sense in their bereavement (54). Being able to make sense of a loss, in terms of understanding the reasons for the death and being able to place the experience within one’s own worldview and belief system, might be an important part of coping with a death in general (55, 56). In the case of genocide in particular, it might be extremely difficult for the bereaved to understand why a loved one was taken from them and to find any sense in it. The fact that a national intention to process and work through the KR period was barely existent in the first decades after the genocide (57–59) presumably adds to individual difficulties to understand how the genocide could have happened. Field and Chhim (38) also found that within a Cambodian sample, those who were not able to find something beneficial in the processing of the experience of loss had a higher wish for revenge.

Besides this aspect, various cognitive components, such as negative cognitions about the self and the future and catastrophic misinterpretations (60) have been shown to be correlates of PGD (61, 62). Boelen, van Denderen (62) showed that not only PTSD and PGD, but also feelings of revenge and anger were linked to several negative cognitions. Moreover, rumination has also been shown to be associated with maladaptive grief reactions and could further hinder the development of a conciliatory attitude (63, 64). A longitudinal study found that rumination about injustice after a loss predicted higher symptom levels of complicated grief (65). It can thus be assumed that the harrowing experience of injustice in the case of murder or even genocide shakes one’s own belief system, triggers negative cognitions and coping styles, and makes adaptive reactions generally more difficult. A study by Tay, Rees (66) also emphasized the link between the sense of injustice and severe grief reactions following human rights violations.

Another important aspect to be considered when looking at attitudes toward reconciliation and PG is the cultural context in Cambodia, especially religious and spiritual beliefs about death and mourning. Research suggests that religious beliefs have the potential to be both a risk (67) and a protective factor (5) for PGD, presumably depending on the ways of coping they offer to the bereaved. During the KR regime, many deaths were violent and unnatural, religion was forbidden and traditional ceremonies after death were not possible (16). For many Cambodians, these aspects may be reasons to believe that a family member was not reincarnated (17). The importance of the lack of Buddhist ceremonies for survivors of the KR regime also becomes apparent in a culturally adapted therapy for KR survivors in which a Buddhist ritual was added to the regular therapy. The ceremony, which was led by monks, was conducted at a mass grave site in Cambodia with the goal of helping the ancestors of the survivors to find a peaceful place (68). Bereaved persons might thus see the KR as responsible for more than the death of a loved one, namely, for having put the deceased in a state in which he or she cannot reincarnate (16). Some persons feel haunted by their relatives, which further connects grief-related symptoms of rumination or nightmares to the deeds of the KR (17–19). Therefore, coming to terms with what has happened and changing the attitude toward the perpetrators might be further complicated by religious and spiritual beliefs about death, causing people to worry about their loved ones even decades after they have died.

Several limitations of the present study should be considered when interpreting the results. First of all, the sample was not recruited through random sampling. Despite the large sample size, the results may thus not be generalizable to all victims of the Khmer Rouge regime. Our measure to assess PG, the CGA-SR, differs from the newly released classification of PGD in the ICD-11 and from the DSM-5 persistent complex bereavement disorder (PCBD) with regard to content and the number of symptoms required to meet diagnosis. The findings of the current study are therefore not generalizable to other definitions of the disorder. Furthermore, the CGA-SR has not been validated for use in Cambodia, although the construct of PGD was validated by Field, Strasser (15) using a similar questionnaire, which speaks for the general applicability of PGD in Cambodia. Moreover, the RRI is a relatively new questionnaire. Although its factor structure has been confirmed in several samples, some caution is warranted when interpreting the results. In particular, the third subscale (ability to differentiate) requires further caution, as it consists only of two items. Finally, the cross-sectional design of the present study does not allow conclusions to be drawn about causal relationships between the assessed variables.

In conclusion, so far, research on post-conflict reconciliation and mental health outcomes has primarily focused on PTSD. However, PG is also very likely to be highly prevalent in post-conflict regions, given the high number of deaths and their mainly unnatural, often violent and man-made nature. The results of the current study suggest that PG should be considered as a mental health-related correlate of reconciliation. As such, the findings can contribute to a better understanding of reconciliation processes in Cambodia and possibly also in other post-conflict regions. The association between attitudes toward reconciliation and PG should therefore be investigated in other post-conflict populations applying contemporary criteria for PGD or PCBD.

## Data Availability Statement

The datasets collected for this study cannot be made publicly available, as we did not obtain consent from our participants for sharing of data when the study was conceived.

## Ethics Statement

The studies involving human participants were reviewed and approved by Universität Konstanz. The participants provided their written informed consent to participate in this study.

## Author Contributions

The study was part of a larger research project on reconciliation and mental health in Cambodia. NS and CK designed the study and wrote the research proposal. NS coordinated the study and supervised the data collection in the field. CK supervised the study. NS, LH, and CH analyzed the data. LH, NS, CH, and MB contributed to the data interpretation and structure of the manuscript. NS and LH drafted the manuscript. All authors contributed to the article and approved the submitted version.

## Funding

The study was funded by the German Ministry of Foreign Affairs (VN06.-320.21/2008/24) and Psychology Beyond Borders for a research project on reconciliation and mental health in Cambodia. The funding agencies were not involved in the design, implementation, analysis, or reporting of the results.

We acknowledge support by the Open Access Publication Initiative of Freie Universität Berlin.

## Conflict of Interest

The authors declare that the research was conducted in the absence of any commercial or financial relationships that could be construed as a potential conflict of interest.
