# Supplementary material for: Association Between Prolonged Grief and Attitudes Toward Reconciliation in Bereaved Survivors of the Khmer Rouge Regime in Cambodia
Source: Front Psychiatry. 2020 Jul 10;11:644. doi: 10.3389/fpsyt.2020.00644 (PMC7366248; doi:10.3389/fpsyt.2020.00644)
Supplement: Supplementary file 1 [file Table_1.pdf]

Supplementary Table 1| Hierarchical Multiple Regression Analysis predicting Openness to Interactions, Absence of Revenge and Differentiation Ability (N = 774)

Outcome	Step	Variables	Model 1		Model 2		Model 3		Model 4	
			**B (SE B)**	***β***	**B (SE B)**	***β***	**B (SE B)**	***β***	**B (SE B)**	***β***
Openness to Interactions[1]	1	Male gender	3.68 (0.45)	0.30***	3.59 (0.46)	0.29***	3.08 (0.46)	0.25***	2.98 (0.46)	0.24***
		Age	-0.05 (0.02)	-0.09**	-0.05 (0.02)	-0.08*	-0.05 (0.02)	-0.09**	-0.05 (0.02)	-0.09**
		Education (years)	0.19 (0.06)	0.11**	0.18 (0.06)	0.11**	0.17 (0.06)	0.10**	0.17 (0.06)	0.10**
	2	Number of losses (close family)			-0.06 (0.06)	-0.03	-0.03 (0.06)	-0.02	-0.01 (0.6)	0.00
		Number of traumatic event types			0.10 (0.05)	0.07*	0.15 (0.05)	0.10**	0.15 (0.05)	0.10**
	3	PTSD symptom severity[2]					-0.09 (0.02)	-0.16***	-0.05 (0.02)	-0.09*
		PG symptom severity[3]							-0.10 (0.03)	-0.12**
R^2 (ΔR^2)			0.141 (0.141***)		0.146 (0.005)		0.170 (0.024***)		0.179 (0.009**)	
Absence of Revenge[1]	1	Male gender	1.06 (0.49)	0.09*	1.06 (0.48)	0.09*	0.18 (0.47)	0.02	-0.01 (0.47)	-0.00
		Age	0.08 (0.02)	0.13***	0.09 (0.02)	0.15***	0.08 (0.02)	0.13***	0.07 (0.02)	0.13***
		Education (years)	0.04 (0.07)	0.02	0.06 (0.07)	0.04	0.04 (0.06)	0.02	0.04 (0.06)	0.02
	2	Number of losses (close family)			-0.22 (0.06)	-0.13***	-0.18 (0.06)	-0.10**	-0.13 (0.06)	-0.08*
		Number of traumatic event types			-0.25 (0.06)	-0.16***	-0.16 (0.05)	-0.10**	-0.16 (0.05)	-0.10**
	3	PTSD symptom severity[2]					-0.15 (0.02)	-0.28***	-0.08 (0.02)	-0.15**
		PG symptom severity[3]							-0.19 (0.03)	-0.24***
R^2 (ΔR^2)			0.026 (0.026***)		0.071 (0.045***)		0.142 (0.071***)		0.176 (0.034)***	
Differentiation Ability[1]	1	Male gender	0.47 (0.13)	0.14***	0.44 (0.13)	0.13**	0.39 (0.14)	0.11**	0.35 (0.14)	0.10*
		Age	0.01 (0.01)	0.03	0.01 (0.01)	0.05	0.01 (0.01)	0.05	0.01 (0.02)	0.05
		Education (years)	0.03 (0.02)	0.07	0.03 (0.02)	0.07	0.03 (0.02)	0.06	0.03 (0.02)	0.06
	2	Number of losses (close family)			-0.05 (0.02)	-0.09*	-0.04 (0.02)	-0.09*	-0.03 (0.02)	-0.07
		Number of traumatic event types			0.01 (0.02)	0.02	0.02 (0.02)	0.03	0.01 (0.02)	0.03
	3	PTSD symptom severity[2]					-0.01 (0.01)	-0.06	0.00 (0.01)	0.02
		PG symptom severity[3]							-0.03 (0.01)	-0.14**
R^2 (ΔR^2)			0.030 (0.030***)		0.038 (0.008*)		0.041 (0.003)		0.054 (0.013**)	

PTSD: Posttraumatic stress disorder, PG: prolonged grief, * p < .05, ** p < .01, *** p < .001 (two-tailed p-values), [1] Subfactor of the RRI, [2] PCL-C, [3] CGA-SR.
